# Locally Sustainable School Lunch Intervention Improves Hemoglobin and Hematocrit Levels and Body Mass Index among Elementary Schoolchildren in Rural West Java, Indonesia

**DOI:** 10.3390/nu9080868

**Published:** 2017-08-12

**Authors:** Makiko Sekiyama, Katrin Roosita, Ryutaro Ohtsuka

**Affiliations:** 1 Graduate Program in Sustainability Science—Global Leadership Initiative, Graduate School of Frontier Sciences, The University of Tokyo, 5-1-5 Kashiwanoha, Kashiwa 277-8563, Japan; 2 Department of Community Nutrition, Faculty of Human Ecology, Bogor Agricultural University, Jl. Lingkar Kampus IPB Darmaga, Bogor 16680, Indonesia; kroosita2@apps.ipb.ac.id; 3 Japan Wildlife Research Center, 3-3-7 Kotobashi, Tokyo 130-8606, Japan; rohtsuka@jwrc.or.jp

**Keywords:** school lunch, child growth, dietary intake, anemia, Indonesia

## Abstract

School lunch is not provided in public elementary schools in Indonesia, and students frequently buy and eat snacks at school. We hypothesized that providing a traditional Sundanese meal as school lunch would be beneficial for children in rural West Java. To test this hypothesis, we evaluated the effect of a 1-month school lunch intervention aiming at sustainability and based on children’s nutritional intake, hemoglobin and hematocrit levels, and body mass index (BMI). A lunch (including rice, vegetable dish, animal protein dish, plant protein dish, and fruit) containing one-third of the recommended daily allowance of energy was offered every school day for 1 month, targeting 68 fourth-grade elementary schoolchildren. At baseline, the prevalence of anemia was 33.3%. The prevalence of stunting and underweight were 32.4% and 2.9%, respectively, whereas that of overweight and obesity combined was 17.6%, indicating a double burden of malnutrition among the subjects. During the intervention, intakes of protein (*p* < 0.05), calcium (*p* < 0.05), and vitamin C (*p* < 0.001) significantly increased, while that of fat significantly decreased (*p* < 0.001). After the intervention, hemoglobin (*p* < 0.05) and hematocrit (*p* < 0.05) levels were significantly improved, thereby almost halving the rate of anemia. These changes were significantly larger in the baseline anemic group than the non-anemic group (*p* < 0.01). BMI significantly increased in the baseline underweight/normal group (*p* < 0.001) but not in the overweight/obese group. The school lunch intervention significantly improved nutritional intakes and health statuses, implying its potential for reducing anemia and resolving the double burden of malnutrition among rural Indonesian schoolchildren.

## 1. Introduction

The recent Indonesian National Basic Health Survey revealed that the prevalence of stunting (height-for-age *z*-score of <−2) and underweight (BMI (body mass index)-for-age *z*-score of <−2) among children aged 5–12 years was 30.7% and 11.2%, respectively, implying that undernutrition remains an unresolved problem among children aged <5 years and school-aged children [1]. According to the same survey, the prevalence of overweight (BMI-for-age *z*-score of 1–2) and obesity (BMI-for-age *z*-score of ≥2) has rapidly increased to 10.8% and 8.0%, respectively [1]. Thus, it is clear that Indonesian school-aged children face a double burden of malnutrition, which is an increasing public health problem in many low- and middle-income countries [2].

To prevent this double burden of malnutrition among school-aged children, a promising approach is to nurture healthy dietary habits during childhood, which is a critical period for growth and development and for the establishment of dietary habits [3]. Therefore, school-based nutrition programs have been increasingly implemented worldwide. For instance, the World Health Organization (WHO) launched the Nutrition-Friendly School Initiative (NFSI) in 2006 to address the double burden of nutrition-related ill health among school-aged children [2]. NFSI promotes the role of the school in caring for children to prevent malnutrition because schools offer various opportunities to educate children and promote their healthy dietary and physical activity habits in cooperation with their parents and community members [4].

In Asia, Japan has the longest history of a national school feeding program, which was implemented under the “School Lunch Act” and enacted in 1954. Under this act, school lunch is regarded as an educational experience to promote the healthy development of the mind and body of schoolchildren [5]. The Japanese school lunch comprises a complete meal and its nutritional value is carefully determined by nutritionists. Similar to Japan, some countries, including Taiwan, India, and Vietnam, began national school feeding programs to promote child health and well-being [6,7,8].

In Indonesia, the government planned a national school feeding program as a community level entry point in the national poverty alleviation strategy of the Sixth Five Year Development Plan in the 1990s. After pilot trials, implementation began in 1996, targeting approximately 2.1 million elementary schoolchildren in >16,000 schools in villages designated as poor according to the Inpres Desa Tertinggal (Presidential Instruction for Villages Left Behind) Program criteria. In this program, a midmorning snack was provided 3 days/week throughout the year or 108 snacks in total per year; each snack contained a minimum of 300 kcal energy and 5 g protein, and the allocated cost for each snack was approximately US $0.10–0.15. The program was expanded to all provinces in 1998, targeting 8.1 million children in 53,000 schools. However, because of the severe Asian economic crisis around 1998, it was soon canceled [9] and since then, there has been no nationwide school feeding program. Meanwhile, Indonesia has been the target of a school feeding program led by the World Food Program—when fortified biscuits were provided to >530,000 children in approximately 3000 primary schools in the most vulnerable areas from 2006 to 2008, hemoglobin levels significantly improved; however, there were no significant improvements in any anthropometric indicators [10].

We conducted nutrition and health surveys in the target village located in West Java, particularly in children. One of the most important findings was that elementary school children frequently bought and ate snacks not only before and after school hours but also during recess in school; such snacking played a crucial role in worsening their nutritional status. In addition, their nutritional status deteriorated simply because the snacks were of very poor nutritional quality [11]. Our analysis of the food typically consumed in the area revealed that the nutrient density, namely the amount of nutrients per unit energy (MJ), of fat and carbohydrate was higher and protein and micronutrients, particularly vitamin C, vitamin A, and calcium, was much lower in snacks (*n* = 68) than in non-snacks (*n* = 153). The ratios of the nutrient density in snacks to that in non-snacks were 1.31 for fat, 1.09 for carbohydrate, 0.54 for protein, 0.43 for iron, 0.25 for phosphorus, 0.07 for calcium, 0.03 for vitamin A, and 0.02 for vitamin C. Furthermore, children’s expenditure on snacks constituted a heavy financial burden for their parents [11]. To improve this situation, we hypothesized that providing traditional Sundanese food, which is highly nutritious, as school lunch instead of consuming snacks would improve student nutritional intake and benefit their health.

In this study, we conducted a 1-month school lunch intervention in a locally sustainable manner, involving the provision of locally available food cooked by villagers, with the advice of nutritionists. Moreover, we considered that the total per-day per-person cost for providing lunch should be equivalent to the per-day per-person expenditure on snacks. This study aimed to clarify the effect of the intervention trial on children’s nutritional status, hemoglobin and hematocrit levels, and BMI.

## 2. Materials and Methods

### 2.1. Study Area and Subjects

The study village Sukajadi is located in the kecamatan (subdistrict) Tamansari, kabupaten (district) Bogor, West Java, Indonesia. Detailed information regarding the study area is reported elsewhere [11,12]. We targeted a public elementary school located in the village.

All 68 subjects were students in the fourth grade at the target elementary school. There were two reasons for selecting fourth-grade students as follows: (1) their adequate memory of the food they had eaten in the past 24 h, and (2), they were in the growth stage before the onset of puberty. Written informed consent was obtained from the parents of each child after explaining the purpose and procedures of the study. The study protocol was approved by the Ethics Committee of the Graduate School of Frontier Sciences, The University of Tokyo.

### 2.2. Research Design

The intervention of providing lunch was performed on every school day for 1 month from August to September 2015. To assess the effect of this intervention, baseline and follow-up surveys were conducted one day before and after the intervention.

**Figure 1:**
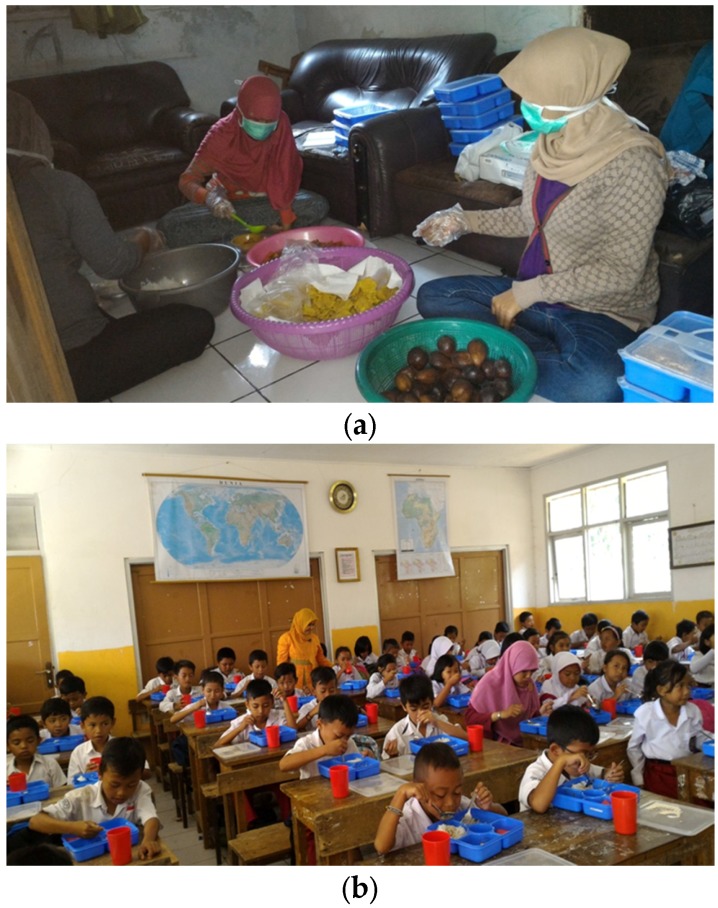
Scenes from our school lunch intervention. (**a**) Village women preparing dishes for lunch. (**b**) Schoolchildren eating lunch.

Given that no school feeding trials had been previously performed in the study area, there was no kitchen or cook at the school. In this study, three local health assistants (called Kader) participated as cooks and the kitchen at the home of one Kader was used for preparing the dishes. Three Kader started cooking at 05:00 and finished around 09:30 and then packed the cooked food in plastic lunchboxes around 11:00 to be carried to the school (Figure 1). The children began eating around 12:00.

The menu for the lunch each day for 1 month was planned by one of the authors (KR), following two principles. One was derived from the nutritional rationality of providing one-third of the energy required per day, based on the Japanese school lunch guidelines by the Ministry of Education, Culture, Sports, Science and Technology [5]. The other was based on the applicability and acceptability of the intervention with respect to the availability of food, workload of cooking, and total cost. As a result, the lunch comprised the following five components: rice, a vegetable dish, an animal protein dish, a plant protein dish, and fruits, all of which are commonly consumed in the area. The total cost, including the cost of food and payment to cooks, per lunch per child was 10,000 Rp (approximately US $0.76).

Because the local tube well was temporarily broken during the intervention period, the students used a water tank, through which spring water was run using a bamboo pipe, for washing their hands. Two nutritionists who were stationed at the school every day instructed the children to wash their hands before eating, to pray just before eating, and to finish the meal within 30 min. The nutritionists also weighed and recorded the leftovers, if any, in each child’s lunchbox.

### 2.3. Data Collection

Intensive data collection was performed twice: in the baseline survey performed 1 day before the intervention and in the follow-up survey performed 1 day after the intervention. In both surveys, we conducted anthropometric measurements, a dietary intake survey, and blood sampling and conducted a questionnaire survey regarding children’s food-related knowledge, behavioral patterns, and socioeconomic features of their households, the data of which are not dealt with in detail in this study. The student’s mother (or caretaker in a few cases) was also invited to attend both baseline and follow-up surveys to assist the child with the dietary intake survey and the questionnaire on socioeconomic features of households.

Anthropometric measurements were conducted by one of the authors (MS), with the assistance of an Indonesian nutritionist. Height was measured to the nearest 1 mm using an anthropometer and body weight was measured with minimum clothing to the nearest 0.1 kg using a Tanita digital weighing scale (Tanita Co., Tokyo, Japan). Mid-upper arm circumference was measured to the nearest 1 mm using a plastic measure tape. Skinfold thickness at the biceps, triceps, and subscapular area were recorded to the nearest 0.1 mm using Eiken calipers (Meikosha Co., Tokyo, Japan). From each child’s height and weight, two *z*-scores (i.e., height-for-age (HAZ) and BMI-for-age (BMIZ)) were calculated based on the WHO growth references published in 2007 [13]. The age of the child was defined by the number of months since his/her birthday. Calculation of *z*-scores, namely HAZ and BMIZ, was performed based on their month-based ages.

Children’s dietary intakes in terms of energy and nutrients were assessed using a 24 h recall method, which was asked to both the child and his/her mother (or caretaker). To guarantee the accuracy of the reported amounts of food consumed as estimated in this 24 h recall procedure, we performed the following steps. First, all snack food available to children at local stores or stalls in and around the schoolyard was weighed. Second, all food (except that in cooked dishes) consumed at home was weighed for various sizes of each foodstuff to determine the “portion size” for each child (e.g., the portion size of rice was determined by weighing the amount in a serving ladle); for food in cooked dishes, we used the “standardized” recipe data based on our previous study in this village [12] to estimate the weight. Then, the consumed amount was estimated from the portion size and number of servings. Finally, the weight of each food consumed by each child was estimated to the nearest gram. Two Indonesian food composition tables [14,15] were used to calculate the amounts of energy and nutrients contained in the food. Nutrient adequacy ratios (NARs) for energy and nutrients were calculated based on the recommended dietary allowance (RDA) for age and sex, according to the following formula [16]:

NAR = Amount of intake by the subject/RDA × 100(1)

A non-fasting 2.0 mL sample of venous blood was obtained from each child by qualified medical staff. Immediately after collection, a small portion was used to measure hemoglobin and hematocrit levels. The remaining portion was aliquoted into a heparinized tube, stored at 4 °C, and centrifuged for 30 min on site. The obtained plasma samples were stored at −20 °C until hormone analysis (equipment for storing samples at −80 °C was unavailable). Capillary tubes, filled with fresh blood and stoppered in the field, were taken to a laboratory at the Bogor Agricultural University. These samples were then centrifuged to determine the hematocrit level. Using 20 μL of blood, hemoglobin levels were determined by the cyanmethemoglobin method. For both hemoglobin and hematocrit, the cut-off values suggested by WHO [17] were used to categorize the children into the anemic or non-anemic group. Children whose hemoglobin or hematocrit level was below the cut-off value were categorized as anemic in this study.

### 2.4. Statistical Analysis

The normality of the distribution of data was examined using the Kolmogorov-Smirnov test. Characteristics of the children were compared between boys and girls using the chi-square test (categorical data), *t*-test (normally distributed data), or Mann–Whitney *U* test (non-normally distributed data). Energy and nutrient intake and hemoglobin and hematocrit levels before and after the intervention were compared using paired *t*-test (normally distributed data) or Wilcoxon signed-rank test (non-normally distributed data) and the anemic status was compared using the chi-square test. Hemoglobin and hematocrit levels were compared using two-way analysis of variance (ANOVA) with repeated measures on two factors, namely ‘baseline anemic status’ (two levels: anemia or non-anemia) and ‘time’ (two levels: before and after the intervention), together with between-factors interaction term. Similarly, BMI and BMIZ levels were compared using two-way ANOVA with repeated measures on two factors, namely ‘baseline BMIZ status’ (two levels: BMIZ < 1 and BMIZ ≥ 1) and ‘time’ (two levels: before and after the intervention), together with between-factors interaction term. Changes in hemoglobin (using Mann-Whitney *U* test, as such data were non-normally distributed) and hematocrit (using *t*-test, as such data were normally distributed) levels before and after the intervention were compared between the anemic and non-anemic groups, as determined in the baseline survey. Statistical significance was defined as *p* < 0.05. All analyses were performed using the Statistical Package for Social Science software package (Version 10.0; SPSS Inc., Chicago, IL, USA).

## 3. Results

### 3.1. Characteristics of the Subjects

Table 1 shows the age and physical characteristics of the children. Their mean age was 114.6 months (9 years and 6 months). The prevalence of stunting was 32.4%, exceeding the national average (30.7%). The prevalence of underweight was 2.9%, being lower than the national average (11.2%), while that of overweight and obese combined was 17.6%, which was similar to the national average (18.8%). For all of these indicators, there was no difference between sexes.

### 3.2. Nutritional Value of the Provided School Lunch

Table 2 shows the means (±SD) of energy and nutrients contained in the provided school lunch; as previously mentioned, the amount of energy was set to be approximately one-third of RDA. Compared with the Indonesian RDA for 7- to 9-year-old children, the provided amount of either energy or protein was 27–29% (or nearly one-third) of the per-day RDA and that of carbohydrate and micronutrients, except calcium, was more than one-third of the per-day RDA, while that of fat and calcium was only 16% and 8%, respectively, of the per-day RDA.

### 3.3. Nutritional Intakes Before and During the Intervention

Table 3 shows the energy and nutrient intakes of the children before and during the intervention, according to the 24 h recall data. The comparison of the values between the two periods using the paired *t*-test (normally distributed data) or Wilcoxon signed-rank test (non-normally distributed data) revealed that the intake of protein, calcium, and vitamin C significantly increased, that of fat significantly decreased, and that of energy and other nutrients, namely carbohydrate, phosphorus, iron, and vitamin A, did not significantly change. When compared with the Indonesian RDA, children’s NAR before and during the intervention correspondingly differed, with the same statistical differences as in the amount of intake. Larger NARs of protein, calcium, and vitamin C during the intervention were of particular interest because they were judged to be less before the intervention, despite their significant roles in human health, particularly healthy growth.

**Figure 2:**
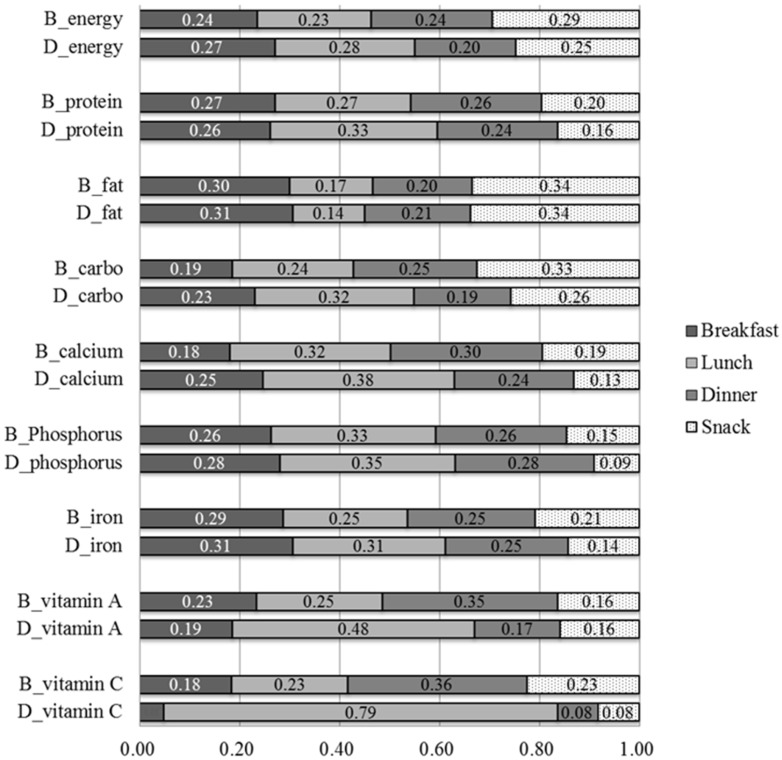
Percentage contributions of breakfast, lunch, dinner, and snacks to the total energy and nutrient intakes before and after the intervention. B_energy or nutrient: intake before the intervention, D_energy or nutrient: intake after the intervention.

Figure 2 illustrates the percentage contributions of breakfast, lunch, dinner, and snacks to the total energy and nutrient intakes before and during the intervention based on the mean values of all children. During the intervention, the percentage contribution of lunch was larger in terms of energy and almost all nutrients except fat, and the differences were particularly prominent for micronutrients, vitamin C, and vitamin A, apparently reflecting their high levels in the provided school lunch.

### 3.4. Hemoglobin and Hematocrit Levels and the Anemic Status Before and after the Intervention

**Figure 3:**
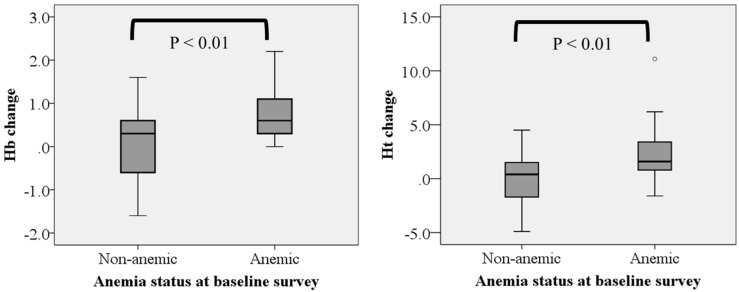
Comparison of changes in hemoglobin and hematocrit levels between anemic and non-anemic groups based on the status at the baseline. The boxes represent the median (black middle line) limited by the 25th (Q1) and 75th (Q3) percentiles. The whiskers are the upper and lower adjacent values, which are the most extreme values within Q3 + 1.5 (Q3 − Q1) and Q1 − 1.5 (Q3 − Q1), respectively. The dot represents an outlier.

Table 4 shows hemoglobin (g/dL) and hematocrit (%) levels based on the baseline anemic status among the children who were properly measured for both baseline and follow-up surveys (*n* = 51 because of failure to sample blood for 17 of them). Both hemoglobin (before intervention, 12.1 ± 1.0; after intervention, 12.4 ± 0.9; *p* < 0.05) and hematocrit (before intervention, 34.9 ± 3.2; after intervention, 35.7 ± 2.5; *p* < 0.05) levels were significantly improved after the intervention. Hemoglobin and hematocrit levels were compared using two-way ANOVA with repeated measures, with ‘baseline anemic status’ (two levels: anemia or non-anemia) and ‘time’ (two levels: before and after the intervention) as the independent valuables. The results showed that there was a significant interaction between baseline anemic status and time (F = 10.363, *p* < 0.01 for hemoglobin, F = 9.338, *p* < 0.01 for hematocrit). There were also main effects of baseline anemic status (F = 29.372, *p* < 0.001 for hemoglobin, F = 46.240, *p* < 0.001 for hematocrit) and time (F = 13.391, *p* < 0.01 for hemoglobin, F = 9.676, *p* < 0.01 for hematocrit). Anemia prevalence was 33.3% at baseline, exceeding the national average (26.4%) [1]. Improvements in hemoglobin and hematocrit levels were observed among 75% and 65% of the children, respectively, and consequently anemia prevalence decreased from 33.3% to 19.6%. Figure 3 shows the changes in hemoglobin (g/dL) and hematocrit (%) levels before and after the intervention when the children were divided into two groups, i.e., anemic and non-anemic groups, as determined by the measurements before the intervention. The difference was significantly larger for hemoglobin and hematocrit levels in the anemic group than in the non-anemic group (*p* < 0.01); in the anemic group, hemoglobin levels improved in all individuals, while hematocrit levels improved in 82% of them.

### 3.5. BMI and BMIZ Before and after the Intervention

**Figure 4:**
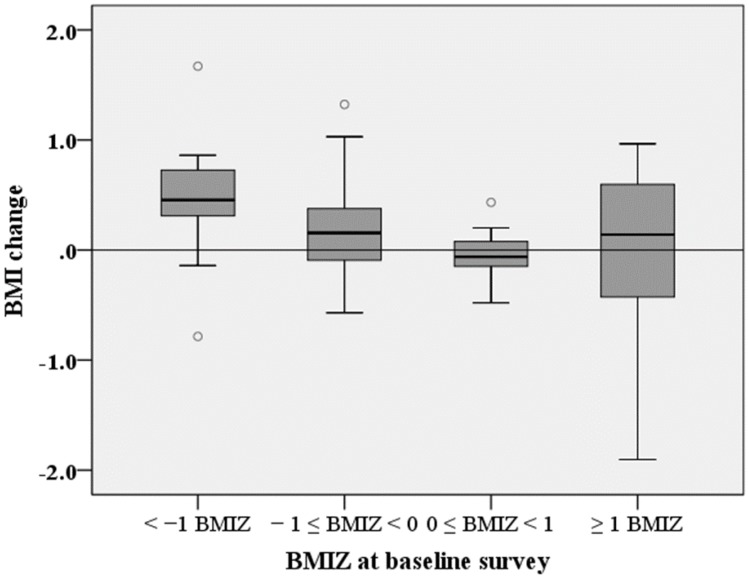
Comparison of body mass index (BMI) change after the 1-month intervention among different BMIZ (*z*-score of BMI-for-age) groups based on the status at the baseline. The boxes represent the median (black middle line) limited by the 25th (Q1) and 75th (Q3) percentiles. The whiskers are the upper and lower adjacent values, which are the most extreme values within Q3 + 1.5 (Q3 − Q1) and Q1 − 1.5 (Q3 − Q1), respectively. The dots represent outliers.

Figure 4 shows the change in BMI of the children before and after the intervention based on the baseline BMIZ status. Table 5 shows BMI and BMIZ of the children before and after the intervention when they were divided into low- (BMIZ < 1, *n* = 54) and high-BMI groups (BMIZ ≥ 1, *n* = 12) based on the measurements before the intervention or baseline. BMI and BMIZ levels were compared using two-way ANOVA with repeated measures, with ‘baseline BMIZ status’ (two levels: BMIZ < 1 and BMIZ ≥ 1) and ‘time’ (two levels: before and after the intervention) as the independent valuables. The results revealed that there was no interaction between baseline BMIZ status and time (F = 1.760, *p* = 1.189 for BMI, F = 3.551, *p* = 0.064 for BMIZ). There was no main effect of time (F = 1.335, *p* = 0.249 for BMI, F = 0.011, *p* = 0.916 for BMIZ), however, there was a significant main effect of baseline BMIZ status (F = 82.877, *p* < 0.001 for BMI, F = 97.190, *p* < 0.001 for BMIZ). In the baseline underweight/normal or low-BMI group, both BMI (*p* < 0.001) and BMIZ (*p* < 0.05) were significantly higher after the intervention than before the intervention. In contrast, in the overweight/obese or high-BMI group, neither BMI nor BMIZ showed a significant difference. Notably, BMI was significantly higher after the intervention when all subjects were pooled.

## 4. Discussion

This is the first trial regarding a school feeding intervention in Indonesia, targeting children in a rural village of West Java. The schoolchildren in the Sukajadi village have been investigated by the authors for >10 years with regard to their health and nutrition statuses [11,12,18]. Similar to many other school-aged children in Indonesia, the subjects faced the double burden of malnutrition, represented by more frequent anemia and stunting and less frequent overweight/obese.

A previous review of school feeding programs in many developing countries worldwide reported four major findings [19]. First, many programs resulted in improving the students’ behavior, as observed in a decrease in the rate of absence from school and progress in scholastic performance [10,20], although there is little evidence regarding the nutritional or health benefits of such programs [21]. Second, the improvement in nutritional intake mainly involved an increase in energy intake [22,23,24]. Third, beneficial effects on the iron status and/or decreased anemia usually correspond to fortification with the associated micronutrients [25,26] and are related to the baseline iron status [25,27]. Fourth, findings on the effects of such programs on physical growth or body composition, represented by body height and weight or BMI, are inconclusive [23,28,29,30].

Our study, based on the provision of locally available Sundanese food as the school lunch at a reasonable cost for 1 month, was designed to investigate the effects of such an intervention on the double burden of malnutrition. There were advantages and disadvantages to this approach. The advantages were the high acceptability of the project among the villagers, particularly the parents of the children, and the change in the food that the children frequently consumed, i.e., from less nutritious snacks to nutritious local food. The disadvantages included the inadequate amounts of calcium contained in local food compared with the Indonesian RDA; during the intervention, the mean per-day calcium intake from all food, including the provided lunch, was 240 mg or only 24% of NAR, despite the mean calcium content being 236 mg/MJ in local non-snacks (*n* = 153) and only 15.9 mg/MJ in snacks (*n* = 68) [11].

There were five main achievements of our intervention trial. First, there was a clear effect on children’s behavior associated with them simply not buying snacks in school during school hours, although their behavior was not a direct focus of the current study. Second, the children’s dietary quality improved during the intervention, while their energy intake did not change. Our baseline survey revealed that one-third of the energy intake was attributable to snacks and the intake of some micronutrients was lower than one-third of RDA before the intervention, indicating the poor quality of the children’s diet because of excessive snacking. The provided school lunch included fruits, vegetables, and animal and protein dishes every day, resulting in improving the protein, calcium, and vitamin C intakes. Such improvements in the diet imply advantageous effects of reducing the prevalence of underweight and anemia. Among the major sources of hemoglobin improvement, namely protein, iron, and vitamin C [25,30], the protein and vitamin C intakes significantly increased during the intervention period. Third, the mean hemoglobin and hematocrit levels increased, decreasing the rate of anemia by half, even without any micronutrient fortification corresponding to the improvement of dietary quality. These increases were prominent in the group with anemia at baseline, all of whose hemoglobin level improved after the 1-month intervention. In the group with anemia at baseline, a significant correlation between vitamin C level increment and hemoglobin level increment was observed (*r* = 0.588, *p* < 0.05), indicating that the improvement of vitamin C intake had a positive effect on the hemoglobin status among children with low baseline iron stores. Fourth, there was a notable result regarding the changes of BMI in children after the 1-month intervention. Considering that 1-month increments of height and weight for 9-year-old children are typically only 0.5 cm and 0.3 kg, respectively [13], it is judged more appropriate to assess the effects of dietary changes on body composition not by the anthropometric measurements themselves but by indicators such as BMI or BMIZ. Among the studies that reported significant improvements in BMI because of school feeding programs in developing countries, only Ahmed et al. obtained dietary data and showed significant increases in energy intake among the subjects [22]; in contrast, our pooled sample showed insignificant changes of energy intake. However, when we divided our sample into groups that were underweight/normal or overweight/obese at baseline, the changes of energy intake between before and during the intervention differed; net change of energy intake of the baseline underweight/normal group was positive (before intervention, 1584 ± 596 kcal; during intervention, 1645 ± 498 kcal), while that of the baseline overweight/obese group was negative (before intervention, 1979 ± 763 kcal; during intervention, 1733 ± 272 kcal). This probably resulted in the different changes in BMI between the baseline underweight/normal and overweight/obese groups. Fifth, this study highlighted the different effects of dietary change on anemic status and body composition between the anemic and non-anemic groups and between the underweight/normal and overweight/obese groups, categorized by measurements at the baseline. Such inter-group differences are beneficial for child nutrition/health programs in many countries, particularly low- and middle-income countries where the double burden of malnutrition has become a serious health issue [2]. Providing meal-based feeding with local dishes is considered to play a significant role in these changes not only for the changes among underweight/anemic children but also for those among overweight/obese ones.

Our study had a few limitations. First, we applied a single 24 h recall method to assess children’s dietary intake, for both baseline and the follow-up surveys. This is despite the fact that several 24 h recalls for the same individual on nonconsecutive days are preferable to assure reproducibility of dietary intake [31]. The main reason stemmed from much high acceptability of the respondents; a single 24 h recall was used in the Indonesian national dietary survey [32]. To enhance precision of the 24 h recall, we interviewed not only the child but also his/her mother (or caretaker). Furthermore, we weighed all foods available to children in and around the schoolyard, visited each student’s home to weigh various sizes of individual food items to determine portion size, and for foods in cooked dishes we used “standardized” recipe data based on our previous study in this village. Second, we failed to study the control group because blood sampling from the children without provision of school lunch in both baseline and follow-up surveys was not ethically accepted in the target community. To partly overcome this limitation, we applied internal comparison in the intervention group—i.e., comparison of hemoglobin/hematocrit change based on baseline anemic status—and comparison of BMI/BMIZ change based on baseline BMIZ status. Third, while existing literature has reported the relationship between hematologic variables and body weight [12,33], we found no such relationship, presumably due to the limited sample size. Fourth, the study was conducted in just one village in West Java, which may challenge the applicability to other villages, or generalization of the survey results. As mentioned, we have conducted nutrition and health surveys in the targeted village since 2000, and based on our previous work we hypothesized that providing traditional Sundanese food as school lunch, in place of snack consumption, would improve children’s nutrition and health. In other words, as the first step, the current study aimed to test our hypothesis by providing school lunch for 1 month, taking the applicability of the results to other areas into consideration. In fact, the Ministry of Education and Culture, Indonesia, gave particular attention to our findings in this study, leading to further implementation of school feeding initiatives in other areas of Indonesia since 2016.

Despite the above-mentioned limitations, the present study provides evidence of school lunch interventions’ efficacy in improving the nutritional and health statuses of school-aged children in Indonesia, as well as other developing countries that face the double burden of malnutrition. As the period of schooling is critical for formation of effective dietary habits, appropriate school feeding programs, in tandem with practicable local food culture, should be promoted.

## Figures and Tables

**Table 1:** Physical characteristics of the children at baseline. HAZ: height-for-age; BMI: body mass index; BMIZ: BMI-for-age.

	*n*	Age (months)	Height (cm)	HAZ	Stunting (≤−2 HAZ)	Weight (kg)	BMI (kg/m^2^)	BMIZ	Underweight (≤−2 BMIZ)	Overweight or Obese (≥1 BMIZ)
		Mean ± SD	Mean ± SD	Mean ± SD	%	Mean ± SD	Mean ± SD	Mean ± SD	%	%
Boy	43	115.2 ± 8.9	125.9 ± 5.4	−1.55 ± 0.81	30.2	26.5 ± 5.9	16.6 ± 2.8	−0.06 ± 1.19	4.7	20.9
Girl	25	113.6 ± 8.5	125.1 ± 6.2	−1.66 ± 0.95	36.0	25.0 ± 4.1	15.9 ± 2.0	−0.37 ± 0.99	0.0	13.6
Both	68	114.6 ± 8.8	125.6 ± 5.7	−1.59 ± 0.86	32.4	25.9 ± 5.3	16.3 ± 2.6	−0.17 ± 1.12	2.9	17.6

Comparisons were performed using the chi-square test (categorical variables), independent *t*-test (normally distributed data), or Mann-Whitney *U* test (non-normally distributed data). No items significantly differed between sexes.

**Table 2:** Mean and SD of energy and nutritional values of the provided school lunch.

	Energy (kcal)	Protein (g)	Fat (g)	Carbohydrate (g)	Calcium (mg)	Phosphorus (mg)	Iron (mg)	Vitamin A (μgRE)	Vitamin C (mg)
Mean	544.8	13.3	11.3	123.6	80.9	532.8	4.5	284.5	29.5
SD	68.4	2.5	4.3	132.8	30.3	745.2	1.8	170.7	21.9
% RDA/day *	29	27	16	49	8	107	45	57	66

* Recommended dietary allowance (RDA) values for calculating the “%RDA/day” were based on a 7- to 9-year-old Indonesian child of 27 kg in weight and 130 cm in height, which is slightly larger than the average of the children in this study.

**Table 3:** Energy and nutrient intakes before and during the intervention by 24 h recall for food consumption (*n* = 68). NAR: nutrient adequacy ratio.

	Before the Intervention		During the Intervention		Difference
	Amount of Intake	NAR (%)	Amount of Intake	NAR (%)	
	(Mean ± SD)		(Mean ± SD)		
Energy (kcal)	1654 ± 640	89.4	1660 ± 465	89.7	ns
Protein (g)	36.7 ± 16.6	75.0	41.7 ± 13.6	85.1	*p* < 0.05
Fat (g)	47.3 ± 26.0	65.6	36.6 ± 15.9	50.9	*p* < 0.001
Carbohydrate (g)	262 ± 98	103.2	298 ± 129	117.4	ns
Calcium (mg)	205 ± 185	20.5	240 ± 155	24.0	*p* < 0.05
Phosphorus (mg)	418 ± 403	83.6	426 ± 207	85.3	ns
Iron (mg)	9.7 ± 5.6	97.2	10.2 ± 4.4	102.2	ns
Vitamin A (μgRE)	300 ± 230	60.0	279 ± 176	55.9	ns
Vitamin C (mg)	12.5 ± 21.5	27.8	64.0 ± 28.0	142.3	*p* < 0.001

Comparison was performed by paired *t*-test (normally distributed data) or Wilcoxon signed-rank test (non-normally distributed data).

**Table 4:** Hemoglobin and hematocrit levels divided into two groups at baseline, before and after the intervention.

	Before Intervention		After Intervention		*p* (Baseline Anemia Status)	*p* (Time)	Interaction
	Baseline Anemia (*n* = 17)	Baseline Non-Anemia (*n* = 34)	Baseline Anemia (*n* = 17)	Baseline Non-Anemia (*n* = 34)			
Hemoglobin (g/dL) ^a^	11.2 ± 0.9	12.6 ± 0.6	11.9 ± 0.9	12.6 ± 0.7	*p* < 0.01	*p* < 0.001	*p* < 0.01
Hematocrit (%) ^a^	31.7 ± 3.0	36.5 ± 1.6	34.0 ± 2.7	36.5 ± 1.9	*p* < 0.01	*p* < 0.001	*p* < 0.01

^a^ Only 51 children were measured properly for hemoglobin and hematocrit levels for both before and after the intervention. Values are means ± SD; two-way ANOVA with repeated measures was used for statistical analysis.

**Table 5:** Body mass index (BMI) and BMIZ (*z*-score of BMI-for-age) of the children, divided into two groups at baseline, before and after the intervention.

	Before Intervention		After Intervention		*p* (Baseline BMIZ)	*p* (Time)	Interaction
	Baseline <1 BMIZ (*n* = 54)	Baseline ≥1 BMIZ (*n* = 12)	Baseline <1 BMIZ (*n* = 54)	Baseline ≥1 BMIZ (*n* = 12)			
BMI (kg/m^2^) ^a^	15.4 ± 1.	20.5 ± 3.4	15.7 ± 0.9	20.5 ± 3.8	*p* < 0.001	ns	ns
BMIZ ^b^	−0.57 ± 0.68	1.70 ± 0.87	−0.47 ± 0.62	1.61 ± 1.01	*p* < 0.001	ns	ns

^a^ Two children were measured properly for height after the intervention. Values are means ± SD; two-way ANOVA with repeated measures was used for statistical analysis.
